# Clinical Characteristics of Cognitive Impairment and 1-Year Outcome in Patients With Anti-LGI1 Antibody Encephalitis

**DOI:** 10.3389/fneur.2020.00852

**Published:** 2020-08-21

**Authors:** Hai-lun Hang, Ji-hong Zhang, Dao-wen Chen, Jie Lu, Jing-ping Shi

**Affiliations:** Department of Neurology, The Affiliated Nanjing Brain Hospital of Nanjing Medical University, Nanjing, China

**Keywords:** anti-LGI1 encephalitis, short-term memory impairment, cognitive outcomes, mini-mental state examination, montreal cognitive assessment-basic

## Abstract

**Introduction:** Anti-leucine-rich glioma-inactivated 1 antibody (anti-LGI1) encephalitis is one of the most common autoimmune encephalitis. Anti-LGI1 encephalitis presented with subacute or acute onset of cognitive impairment, psychiatric disturbances, faciobrachial dystonic seizures (FBDSs), convulsions, and hyponatremia. The common sequela of anti-LGI1 encephalitis is cognitive disorder, but there are few studies on the recovery of cognitive function after immunotherapy. This study aimed to explore clinical characteristics of cognitive impairment and 1-year outcome in patients with anti-LGI1 encephalitis.

**Methods:** The clinical data and characteristics of cognitive impairment of 21 patients with anti-LGI1 encephalitis from 2016 to 2019 in Nanjing Brain Hospital were analyzed retrospectively. At the time of onset of hospitalization and 1 year after discharge, the cognitive functions in these patients were assessed using two cognitive screening scales—Mini-Mental State Examination (MMSE) and Montreal Cognitive Assessment-Basic (MoCA-B).

**Results:** Among the 21 patients, 13 were male and 8 were female, aged 51.10 ± 14.69 (age range 20–72) years. Nineteen patients, comprising 90.48%, had recent memory deterioration. Routine electroencephalography (EEG) results of 13 cases were abnormal. EEG results were epileptic or slow-wave activity involving the temporal lobes. Eleven cases of brain MRI were abnormal, and the focus involved the hippocampus and mediotemporal lobe. The decrease of short-term memory [recall scores: 0.57 ± 0.81 (MMSE), 0.76 ± 1.34 (MoCA-B)] is the most obvious at the time of admission. After intravenous (IV) injection of methylprednisolone and/or immunoglobulin, the clinical symptoms of the patients improved obviously. Total MMSE and MoCA-B scores of patients were significant increased after 1 year (21.19 ± 3.54 vs. 26.10 ± 3.02, *P* < 0.001; and 19.00 ± 4.38 vs. 25.19 ± 4.25, *P* < 0.001, respectively). Recall scores and orientation scores of MoCA-B were significantly improved after 1 year (0.76 ± 1.34 vs. 3.24 ± 1.48, *P* < 0.001; and 3.10 ± 1.26 vs. 5.00 ± 1.22, *P* < 0.001, respectively). However, 3/21 (14.29%) patients still have obvious short-term memory impairment (recall scores ≤ 1).

**Conclusion:** Cognitive impairment is one of the most common manifestations of anti-LGI1 encephalitis, with the main prominent being acute or subacute short-term memory loss. Although most patients with anti-LGI1 encephalitis respond well to immunotherapy, a small number of patients still have cognitive disorders, mainly recent memory impairment, after 1 year.

## Introduction

Autoimmune encephalitis (AE) is a rare and newly discovered inflammation disease (1–6) of the nervous system, which is related to specific autoantibodies (Abs). Among them, anti-LGI1 encephalitis (2) is a treatable etiology of AE. LGI1-Abs were found in 2010 (3), which may be the second most common cause of AE following anti-*N*-methyl-d-aspartate receptor (NMDAR) encephalitis and the most common cause of limbic encephalitis (LE) (4–6). The common manifestations of anti-LGI1 encephalitis are cognitive impairment or rapidly progressive dementia (7), psychiatric disturbances, convulsions (2, 8), faciobrachial dystonic seizures (FBDSs), and refractory hyponatremia (7). Anti-LGI1 encephalitis typically evolves and predominately affects middle-aged and elderly males over 50 years old (8, 9). Anti-LGI1 encephalitis has a good response to hormone and other immune system-based therapy (8, 9).

Cognitive impairment could be seen in most patients with anti-LGI1 encephalitis, and it is often (10), predominately, memory deterioration. It is reported (9–11) that about 25% of patients have complete recovery of cognitive function, whereas in others, mild disability may be a persistent sequela of the disease. There are more and more reports of patients with anti-LGI1 encephalitis (7–11); however, the characteristics of cognitive impairment in patients among the Chinese population with anti-LGI1 encephalitis have not been described.

The Mini-Mental State Examination (MMSE) is the gold standard of cognitive assessment for adults and the elderly. The MMSE has been proven to be effective and reliable in clinical and research settings, including adult, geriatric, hospital, and residential environments. The MMSE is the most extensively and widely validated tool (12) for cognitive assessment. The MoCA is a cognitive screening tool similar to MMSE, which pays more attention to and executive function of the frontal lobe. Montreal Cognitive Examination-Basic (MoCA-B) (13) is an improved version of MoCA, especially for the elderly subjects. MMSE and MoCA-B tests enable health-care providers to quickly assess patients' cognitive health and accurately make more informed medical decisions. MMSE and MoCA are two commonly used tools to measure cognitive impairment. A few studies have reported (9, 10) their application in AE cognitive assessment. In this study, the MoCA-B was used to compare the scores obtained by subjects to MMSE scores. The aim of this study is to characterize the clinical presentation and 1-year outcome, especially cognitive impairment in patients with anti-LGI1 encephalitis.

## Methods and Materials

### Patients and Laboratory Tests, Electroencephalography, and Imaging Examination

This was an observational study conducted from January 2016 to December 2019 on hospital inpatients at the Affiliated Brain Hospital of Nanjing Medical University, China. We reviewed 21 patients who were diagnosed with anti-LGI1 encephalitis. All patients underwent a series of laboratory tests, including standard biochemistry, viral Abs (including herpes simplex virus 1 and 2, and herpes zoster virus), syphilis, HIV, thyroid function, rheumatic indicators, tumor biomarkers, and AE-related Abs [NMDAR, LGI1, GABABR, contactin-associated protein-like 2 (CASPR2), AMPA1R, and AMPA2R, and classical paratuberculosis Abs, such as Hu, Ri,Yo, Ma2, amphiphysin, CV2, ANNA-3, PCA-2, and Trand GAD], as well as other laboratory tests. Autoimmune encephalitis-related Abs of these patients also received a cerebrospinal fluid (CSF) test. The blood and CSF AE-related Abs were tested with commercial kits (Euroimmun, Germany) by indirect immunofluorescence testing (IIFT) as we previously described (6). All the 21 patients underwent chest CT, abdominal ultrasonography, brain magnetic resonance imaging (MRI), and routine electroencephalography (EEG) examinations. Clinical data from 21 patients who were diagnosed with anti-LGI1 encephalitis were collected and analyzed.

### Clinical Evaluations

The MMSE and MoCA-B are routinely administered in our Department of Neurology. Both the MMSE and MoCA-B were conducted on the same day by a trained clinical psychologist. The MMSE and MoCA-B scores were used to assess the cognitive function of each patient at the time of early onset (the MMSE and MoCA-B collected within 1 week after admission) and at the time of follow-up 1 year later (the MMSE and MoCA-B collected 15 days before discharge and 1 year after discharge).

The MMSE scale consists of 30 questions, and the highest score is 30 points. Higher scores indicate better cognition. The MMSE tests five cognitive domains: time and place orientation (10 points), memory registration (3 points) and recall (3 points), attention and calculation (5 points), and language and praxis (9 points). MoCA-B measures nine cognitive domains including executive (1 point), abstraction (3 points), recall (5 points), verbal fluency (2 points), visuospatial (3 points), orientation (6 points), naming (4 points), calculation (3 points), and attention (3 points). The MoCA-B score is between 0 and 30, the higher the score, the better the cognitive function. Subjects who scored 27 or more on MMSE and MoCA-B (14, 15) were considered cognitively normal; those with MMSE score of 21–26 and MoCA-B score of 18–26 suffer from mild cognitive impairment; those with MMSE score of 10–20 and MoCA-B score of 10–17 suffer from moderate cognitive impairment; and those with 9 points or less suffer from severe cognitive impairment. All subjects were assessed with the MMSE and the MoCA-B in addition to the required radiological and laboratory examinations.

### Treatment

During hospitalization, all patients accepted first-line immune therapy [intravenous (IV) methylprednisolone], and 16 (76.19%) were treated with combination of IV methylprednisolone and immunoglobulins. The regimen was prednisolone at an initial dose of 60 mg daily, tapering (5 mg/half a month) within half a year until total withdrawal. Fifteen of 19 anti-LGI1 encephalitis patients were treated with chronic immunotherapy [mycophenolate mofetil (MMF)]. This study was approved by the Ethics Committee of the Affiliated Brain Hospital of Nanjing Medical University in accordance with the Declaration of Helsinki. Written informed consent was obtained from all patients.

### Statistical Analysis

Data of MMSE and MoCA-B scores were represented as mean ± SD and were examined for the homogeneity of variance. The paired samples *t*-test was used to compare the differences of MMSE and MoCA-B scores at symptom onset and after 1-year treatment. Correlations between serum and CSF anti-LGI1 Ab titers, and MMSE and MoCA-B scores were evaluated using a Pearson's correlation coefficient. *P* < 0.05 was considered statistically significant. All statistical analyses were performed using SPSS version 16.0 software.

## Results

### Demographic Data and Clinical Features

Among the 21 patients, 13 were male and 8 were female, aged 51.10 ± 14.69 (age range 20–72) years (Table 1). These patients had 11.76 ± 2.96 years of education. Interval from symptom onset of the disease to this admission was 44.67 ± 64.98 days and ranged from 5 for 270 days. Nineteen patients, comprising 90.48%, had recent memory deterioration; 15 (71.43%) patients had dysphrenia; 13 (61.90%) patients had hyponatremia; 15 (71.43%) patients had epileptic seizures; and 11 (52.38%) patients had FBDS. Routine EEG results of 13 cases were abnormal. EEG results were epileptic or slow-wave activity involving the temporal lobes. The brain MRI findings of 11 cases were abnormal, and the lesions involved the hippocampus and mediotemporal lobe. Two patients had tumor (one was thymoma and the other was an adrenal space-occupying lesion). LGI1 Ab was positive in the serum of 20 patients. LGI1 Ab was positive in CSF of 18 patients. Both serum and CSF LGI1 Abs of 17 patients were positive.

**Table 1:** Demographic data and patient characteristics.

**Demographic data and characteristic of the patients**	
Age at onset, mean ± SD (range) (years)	51.10 ± 14.69 (20–72)
Male, *n* (%)	13, 61.90%
Education (years)	11.76 ± 2.96
Time from onset to diagnosis (range) (days)	44.67 ± 64.98 (5–270)
Memory decline, *n* (%)	19 (90.48%)
Seizure, *n* (%)	15 (71.43%)
Dysphrenia, *n* (%)	15 (71.43%)
Hyponatremia, *n* (%)	13 (61.90%)
FBDS, *n* (%)	11 (52.38%)
Tumor, *n* (%)	2 (9.5%)
Abnormal EEG, *n* (%)	13 (61.90%)
Abnormal brain MRI, *n* (%)	11 (52.38%)
Positive antibodies to LGI1 (Serum), *n* (%)	20 (95.24%)
Positive antibodies to LGI1 (CSF), *n* (%)	18 (85.71%)
Double positive to LGI1 (serum + CSF), *n* (%)	17 (80.95%)

*n, number of patients; FBDS, faciobrachial dystonic seizures; CSF, cerebrospinal fluid; LGI1, leucine-rich glioma-inactivated 1; MRI, magnetic resonance imaging*.

### Brain MRI

**Figure 1:**
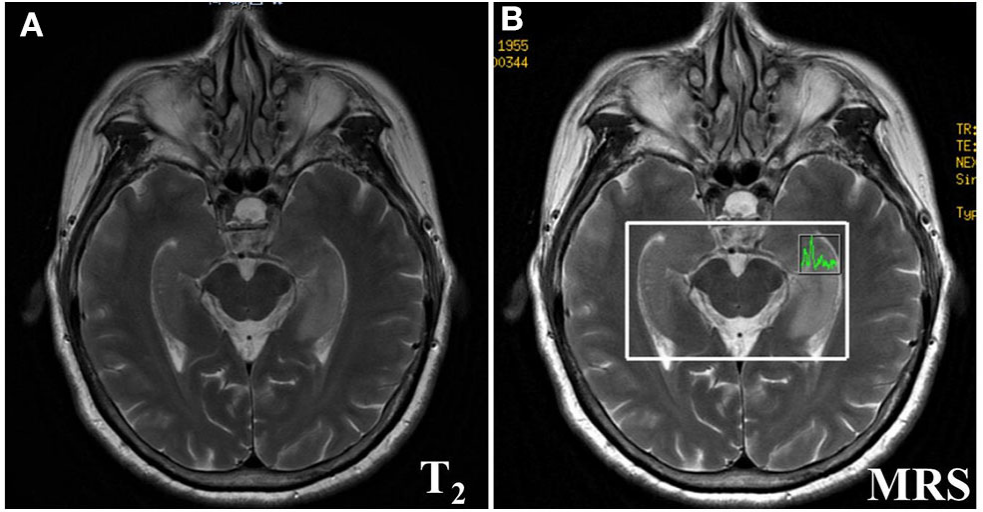
Brain MRI **(A)** showed lesions in the left hippocampus. Brain Magnetic resonance spectrum (MRS) showed a bit increased slightly elevated Choline compound (Cho) and Cho/*Cr* peak, the moderately decreased the N-acetyl aspartic acid (NAA) and NAA/Creatine (*Cr)* peak **(B)** in the left hippocampus.

**Figure 2:**
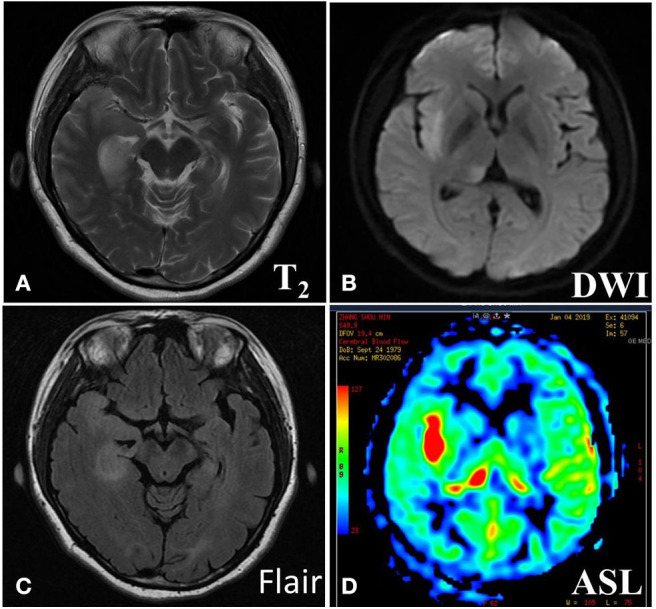
Brain MRI **(A)** showed abnormal signal in right temporal and insular lobe, thalamus. On T2WI **(B)** and T2Flair **(C)** sequences, the right temporal and insular lobe, right thalamus showed slightly higher abnormal signal, the local cortex was slightly swollen, and on the DWI sequences, slightly higher signal was seen. On T2Flair sequence **(C)**, there was high abnormal signal in the right hippocampus and no obvious abnormal signal in the left hippocampus. Arterial spin labeling (ASL) sequence **(D)** showed significant hyperperfusion in the right temporal and insular lobe, thalamus.

The imaging manifestations of anti-LGI1 encephalitis patients mainly were high T_2_ signal and fluid-attenuated inversion recovery (FLAIR) in the bilateral temporal lobe. Some patients had an abnormal signal in one or both sides of the hippocampus region. In addition, the basal ganglia region and temporal lobe are often involved. In this study, MRIs were abnormal in 11 (52.38%) of the 21 cases. The most common lesions involved the hippocampus and temporal lobe. Brain MRI in most patients with anti-LGI1 encephalitis shows a hyperintense signal in the unilateral or bilateral medial temporal lobes. Brain MRI (Figure 1) showed an abnormal signal in the left hippocampus region. Brain magnetic resonance spectroscopy (MRS) showed moderately decreased *N*-acetyl aspartic acid (NAA) and NAA/creatine (Cr) peak, and slightly elevated choline compound (Cho) and Cho/Cr peak. Brain MRI (Figure 2) showed the focus in the right temporal and insular lobes and the thalamus. On T_2_WI and T_2_FLAIR sequences, the right temporal and insular lobes and the right thalamus showed a slightly higher abnormal signal; and on the diffusion-weighted imaging (DWI) sequences, a slightly higher signal was seen. On T_2_FLAIR sequence, there were abnormal hyperintensities in the right hippocampus and no obvious abnormal signal in the left hippocampus. Arterial spin labeling (ASL) showed significant hyperperfusion in the right temporal and insular lobes and the thalamus.

### The Mini-Mental State Examination Scores

**Figure 3:**
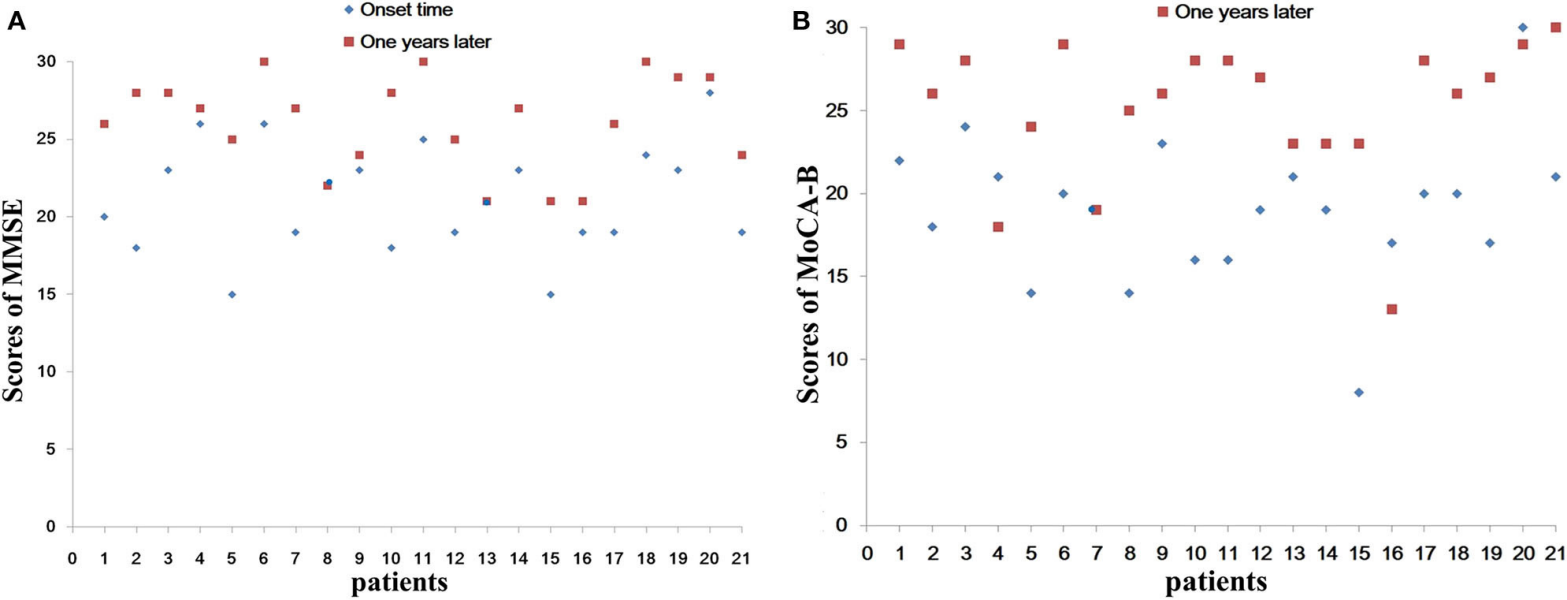
Total scores of MMSE **(A)** and MoCA-B **(B)** of different patients. We separately list the different patient with total scores of MMSE and MoCA-B at the time of hospitalization and 1 year after discharge. **(A)** Show the distribution of the MMSE scores for the 21 cases. **(B)** Show the distribution of the MoCA-B scores for the 21 cases. No moderate to severe cognitive impairment (MMSE ≤ 20) was determined at 1 year **(A)**. MMSE, Mini-Mental State Examination; MoCA-B, Montreal Cognitive Assessment-Basic.

MMSE is the most commonly used cognitive function screening tool. Figure 3A and Table 2 show the distribution of the MMSE scores for the 21 cases at the time of hospitalization and 1 year after discharge. Meanwhile, we counted the improvement of scores mean ± SD [95% confidence interval (CI)] (Table 2). Total MMSE scores of patients were significantly increased after 1-year follow-up (21.19 ± 3.54 vs. 26.10 ± 3.02, *P* < 0.001). After 1 year, the MMSE score of the patients improved by 4.90 ± 3.18 (3.46–6.35) compared with that of the patients at the time of onset, and the difference was statistically significant (*P* < 0.001). No moderate-to-severe cognitive impairment (MMSE ≤ 20) was determined at 1 year (Figure 3A).

**Table 2:** Domains of MMSE test in patients with anti-LGI1 antibody encephalitis.

**MMSE**	**Onset time**	**1 year later**	**Improved score (95% CI)**	***P***
Orientation (10 points)	6.86 ± 1.82	9.14 ± 1.01	2.57 ± 1.66 (1.46–3.11)	<0.001^※^
Registration (3 points)	2.43 ± 0.51	2.71 ± 0.46	0.29 ± 0.56 (0.03–0.54)	0.03^※^
Attention and calculation (5 points)	4.33 ± 0.58	4.57 ± 0.51	0.14 ± 0.57 (−0.12–0.40)	0.09
Recall (3 points)	0.57 ± 0.81	2.14 ± 0.96	1.57 ± 0.81 (1.08–2.06)	<0.001^※^
Language and praxis (9 points)	7.00 ± 1.09	7.90 ± 1.09	0.90 ± 1.04 (0.43–1.38)	0.01^※^
Total (30 points)	21.19 ± 3.54	26.10 ± 3.02	4.90 ± 3.18 (3.46–6.35)	<0.001^※^

*MMSE, Mini-Mental State Examination; LGI1, leucine-rich glioma-inactivated 1*.

※ *Statistically significant value*.

**Figure 4:**
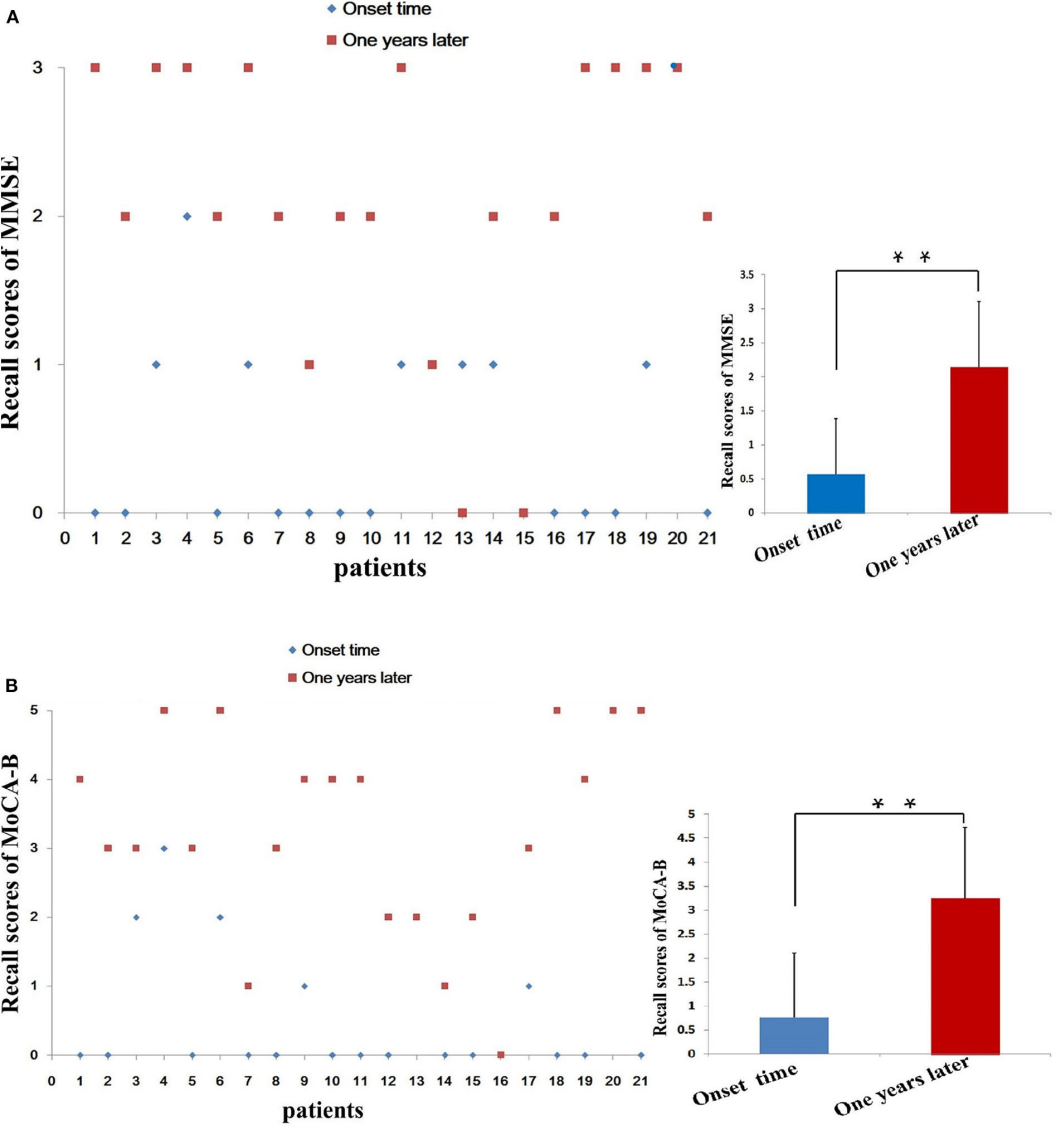
Distribution of patients by short-term memory (recall) score. We separately list the distribution of patients with different recall scores of MMSE **(A)** and MoCA-B **(B)**. The MMSE recall items of 12 (57.14%) patients at the time of onset were scored 0 points, while only 2 patient after 1 year were scored 0 points. Only 1 patient had a normal recall of 3 points during the onset, and 9 patients got 3 points in this item after 1 year. MMSE, Mini-Mental State Examination; MoCA-B, Montreal Cognitive Assessment-Basic; **P < 0.01.

Because the decrease of short-term memory (recall scores: 0.57 ± 0.81) is the most obvious at the time of admission (Figure 3A), we separately listed the distribution of patients with different recall scores of MMSE (Figure 4A). The MMSE recall items of 12 (57.14%) patients were scored 0 points at the time of onset, whereas only two patients were scored 0 points after 1 year. Only one patient had a normal recall of 3 points during the onset, and nine patients had 3 points in this item after 1 year. Orientation, registration, recall, and language scores of MMSE were significantly improved after 1-year follow-up (6.86 ± 1.82 vs. 9.14 ± 1.01, *P* < 0.001; 2.43 ± 0.51 vs. 2.71 ± 0.46, *P* = 0.03; 0.57 ± 0.81 vs. 2.14 ± 0.96, *P* < 0.001; and 7.00 ± 1.09 vs. 7.90 ± 1.09, *P* = 0.01, respectively). One year after immunotherapy, the patients' clinical symptoms improved obviously; however, 4/21 (19.05%) patients still have obvious short-term memory impairment (recall scores ≤ 1) (Figure 4A).

### The MoCA-B Scores

Figure 3B and Table 3 show the distribution of the MoCA-B scores for the 21 cases at the time of hospitalization and 1 year after discharge. Meanwhile, we counted the improvement of scores mean ± SD (95% CI) (Table 3). Total MoCA-B scores of patients were significant increased after 1-year follow-up (19.00 ± 4.38 vs. 25.19 ± 4.25, *P* < 0.001). After 1 year, the MoCA-B score of the patients improved by 6.19 ± 5.19 (3.83–8.55) compared with that of the patients at the time of onset, and the difference was statistically significant (*P* < 0.001). Moderate-to-severe cognitive impairment (MoCA-B ≤ 20) was determined in one of 21 patients at 1 year (Figure 3B).

**Table 3:** Domains of MoCA-B test in patients with anti-LGI1 antibody encephalitis.

**MoCA-B**	**Onset time**	**1 year later**	**Improved score**	***P***
Executive (1 point)	0.76 ± 0.44	0.90 ± 0.30	0.14 ± 0.48 (−0.07–0.36)	0.17
Abstraction (3 points)	2.43 ± 0.60	2.71 ± 0.46	0.29 ± 0.72 (−0.04–0.61)	0.08
Recall (5 points)	0.76 ± 1.34	3.24 ± 1.48	2.48 ± 1.57 (1.76–3.19)	<0.001^※^
Verbal fluency (2 points)	1.57 ± 0.60	1.86 ± 0.36	0.29 ± 0.72 (−0.04–0.61)	0.08
Visuospatial (3 points)	2.38 ± 0.59	2.62 ± 0.59	0.24 ± 0.62 (−0.05–0.52)	0.10
Orientation (6 points)	3.10 ± 1.26	5.00 ± 1.22	1.90 ± 1.48 (1.23–2.58)	<0.001^※^
Naming (4 points)	3.38 ± 0.80	3.62 ± 0.74	0.24 ± 0.94 (−0.19–0.67)	0.26
Calculation (3 points)	2.29 ± 0.85	2.57 ± 0.68	0.29 ± 1.06 (−0.19–0.77)	0.23
Attention (3 points)	2.33 ± 0.66	2.67 ± 0.58	0.33 ± 0.80 (−0.03–0.70)	0.07
Total (30 points)	19.00 ± 4.38	25.19 ± 4.25	6.19 ± 5.19 (3.83–8.55)	<0.001^※^

*MoCA-B, Montreal Cognitive Assessment-Basic; LGI1, leucine-rich glioma-inactivated 1*.

※ *Statistically significant value*.

Because the decrease of short-term memory (recall scores: 0.76 ± 1.34) is the most obvious at the time of admission, we separately list the distribution of patients with different recall scores of MMSE and MoCA-B (Figure 4). The MoCA-B recall items (Figure 4B) of 14 (66.67%) patients at the time of onset were scored 0 points, whereas those of only one patient after 1 year were scored 0 points. Only one patient had a normal recall of 5 points during the onset, and five patients had 5 points in this item after 1 year. Recall scores (0.76 ± 1.34) and orientation scores (3.10 ± 1.26) of MoCA-B decreased significantly at the symptom onset (Table 3). Recall scores and orientation scores of MoCA-B were significantly improved after 1-year follow-up (0.76 ± 1.34 vs. 3.24 ± 1.48, *P* < 0.001; and 3.10 ± 1.26 vs. 5.00 ± 1.22, *P* < 0.001, respectively). One year after immunotherapy, the patients' clinical symptoms improved obviously; however, 3/21 (14.29%) patients still have short-term memory impairment (recall scores ≤ 1) (Figure 4). There were three patients with poor cognitive function recovery in the current study. The three patients were diagnosed as anti-LGI1 encephalitis at 4, 5, and 9 months after onset and then immunotherapy. The time from onset to diagnosis was significantly delayed compared with that of most patients (average range: 44.67 ± 64.98 days) (Table 1). One patient was diagnosed with anti-LGI1 encephalitis 9 months after the onset of the disease, and brain MRI showed hippocampal atrophy. Because the effect of cognitive decline was not obvious, the immunotherapy was not continued after discharge.

### Anti-LGI1 Antibody Titers and Cognitive Scores

No correlation was found between MMSE scores and serum anti-LGI1 Ab titers (*n* = 20) during the onset (*r* = −0.139, *P* = 0.559) or after 1 year (*r* = 0.118, *P* = 0.621). No correlation was found between MoCA-B scores and serum anti-LGI1 Ab titers (*n* = 20) during the onset (*r* = 0.042, *P* = 0.860) or after 1 year (*r* = −0.099, *P* = 0.677). There was no statistically significant correlation between MMSE scores and CSF anti-LGI1 Ab titers (*n* = 18) during the onset (*r* = 0.095, *P* = 0.707) or after 1 year (*r* = 0.159, *P* = 0.529). There was no statistically significant correlation between MoCA-B scores and CSF anti-LGI1 Ab titers (*n* = 18) during the onset (*r* = −0.419, *P* = 0.083) or after 1 year (*r* = −0.066, *P* = 0.796). There was no significant correlation between the serum and CSF Ab titer and the prognosis of cognitive impairment.

## Discussion

Autoimmune encephalitis accounts for 10–20% of cases of encephalitis (1), with anti-NMDAR encephalitis being the most common, accounting for about 80% of AE patients (6), followed by anti-LGI1 encephalitis and anti-γ-aminobutyric acid type B receptor (GABA_B_R) Ab-related encephalitis. Anti-LGI1 Ab, anti-GABA_B_R, and anti-AMPAR Ab-associated encephalitis mainly involve the limbic system and are called autoimmune LE (16). LE is an AE involving the limbic system, including the medial temporal lobe, amygdala, hippocampus, cingulate cortex, and insular lobe. LE is considered to be a disease associated with epilepsy, memory deterioration, and psychobehavioral disorders. LE is associated with Abs to the voltage-gated potassium channel complex (VGKC), and Abs mainly point to the VGKC-complex proteins, CASPR2, or LGI1 protein (17). The Abs involved are mostly LGI1, which is an anti-neuronal surface Ab, accounting for 30% (18) of LE-related Abs. LGI1 is mainly a non-malignant tumor (9–11) and thought to be responsive to immunotherapy.

The clinical manifestations of anti-LGI1 encephalitis are various. Cognitive impairment is the most common manifestation. Memory disorders, especially near memory disorders, are the most prominent (9–11, 19). Some researchers (19, 20) have confirmed that cognitive impairment was related to the course of disease before immunotherapy. Among the cases we studied, one case was diagnosed as anti-LGI1 encephalitis at the time of 9 months after the onset of the disease, and the effect of immunotherapy was poor. The MoCA and MMSE tests were done in all the 21 patients, and the results found that 19 patients, comprising 90.48%, suffered from memory deterioration. EEG and MRI were consistent with involvement of the limbic system. Figures 1, 2 show the abnormal MRI signals in the bilateral or unilateral hippocampus. This indicated that the abnormal signal changes in the hippocampus may be related to memory impairment of the patients.

In 2010, Irani et al. (3) first discovered that LGI1 Ab was involved in the pathogenesis of AE. Anti-LGI1 encephalitis is more common (8) in middle-aged and elderly males (over 50 years of age). In the current study, 13 were male and 8 were female, aged 51.10 ± 14.69 (age range 20–72) years. Most of them have acute or subacute cognitive disorder. Anti-LGI1 encephalitis is the main type of autoimmune LE, generally (7) associated with rapidly progressing cognitive impairment. The main symptoms (8–11) were episodic memory impairment, temporal lobe seizures, asymmetric FBDS, and mental behavior abnormalities. As the most common type of VGKC-Ab encephalitis, cognitive disorders are common in anti-LGI1 encephalitis. In our study, 19 patients, comprising 90.48%, had recent memory deterioration.

Although there is no clear standardized treatment, immunotherapy, including first-line drugs (21)—IV methylprednisolone, plasma exchange, IV immunoglobulin, and other immune support—is strongly recommended. All 21 patients accepted first-line immune therapy (IV methylprednisolone), and 16 (76.19%) patients were treated with combination of IV methylprednisolone and immunoglobulin. The regimen was prednisolone at an initial dose of 60 mg daily after discharge, tapering (5 mg/half a month) within half a year until total withdrawal. Fifteen of 19 anti-LGI1 encephalitis patients were treated with chronic immunotherapy MMF 750 mg twice daily. After immunotherapy, the clinical symptoms of all 21 patients were improved in varying degrees. One year later, cognitive function had also been improved significantly (Tables 2, 3). In the early stage, the patients are given the treatments of IV; in particular, immunoglobulin combined with hormone therapy is better than glucocorticoid alone (21, 22). At present, most patients with anti-LGI1 encephalitis have a relatively good prognosis after immunotherapy. FBDS can be quickly relieved, and most symptoms can be improved; however, cognitive status is slowly improved, and some patients (23) may have permanent memory impairment. The better understanding will be of great significance for early diagnosis, essentially immunotherapy, and even better prognosis. Some studies suggest (9–11, 23) that effective and long-term immunotherapy should be given to prevent long-term complications, including hippocampal atrophy (23) and sustained memory impairment (13). Second-line drugs can be added to the therapy of patients (23, 24) who did not respond well to the first-line drugs or had a recurrence, including rituximab, MMF, or cyclophosphamide. Once cognitive impairment is confirmed, patients should receive immunotherapy (9–11) and long-term maintenance therapy to relieve their symptoms (23, 24), improve prognosis, and avoid intractable epilepsy and hippocampal atrophy. Anti-LGI1 Ab encephalitis may recur or become chronic, as well as legacy cognitive sequelae.

In our current study, the decrease of short-term memory [recall scores: 0.57 ± 0.81 (MMSE), 0.76 ± 1.34 (MoCA-B)] is the most obvious at the time of admission. After the combined treatment of IV methylprednisolone and immunoglobulins, the patients' clinical symptoms improved obviously. Total MMSE and MoCA-B scores of patients at symptom onset were significant increased after 1 year (21.19 ± 3.54 vs. 26.10 ± 3.02, *P* < 0.001; and 19.00 ± 4.38 vs. 25.19 ± 4.25, *P* < 0.001, respectively). Recall scores of MMSE and MoCA-B were significantly improved after 1-year follow-up (0.57 ± 0.81 vs. 2.14 ± 0.96, *P* < 0.001; and 0.76 ± 1.34 vs. 3.24 ± 1.48, *P* < 0.001, respectively). However, 3/21 (14.29%) patients still have obvious short-term memory impairment (MoCA-B recall scores ≤ 1). The common sequela of anti-LGI1 encephalitis is cognitive impairment, especially recent memory impairment. Therefore, it is more necessary to add long-term immunotherapy including MMF to the first-line immunotherapy.

### Limitations and Conclusions

Cognitive impairment is one of the most common manifestations of anti-LGI1 encephalitis, with the main prominent being acute or subacute short-term loss. The MMSE and MoCA-B scales can be used to evaluate cognitive function in patients with anti-LGI1 encephalitis. Although most patients of anti-LGI1 encephalitis had a good cognitive outcome, a small number of patients still have cognitive impairment, mainly short-term memory loss after 1 year. Early and long-term effective immunotherapy of anti-LGI1 encephalitis (10, 11, 20) can obtain better cognitive functional prognosis, so early diagnosis and early treatment of this disease are recommended.

## Data Availability Statement

The raw data supporting the conclusions of this article will be made available by the authors, without undue reservation.

## Ethics Statement

The studies involving human participants were reviewed and approved by the Ethics Committee of the Affiliated Brain Hospital of Nanjing Medical University. The patients/participants provided their written informed consent to participate in this study. Written informed consent was obtained from the individual(s) for the publication of any potentially identifiable images or data included in this article.

## Author Contributions

HH, JL, and JS conceived and designed the study. HH and JL analyzed the data. HH and JL drafted the manuscript. HH, JZ, DC, JL, and JS critically reviewed the manuscript. All authors contributed to the article and approved the submitted version.

## Conflict of Interest

The authors declare that the research was conducted in the absence of any commercial or financial relationships that could be construed as a potential conflict of interest.
